# Usefulness of Glucose Measurement To Assess Tissue Perfusion of the Nipple-Areola Complex in Conservative Mastectomy: A Prospective Cohort Study Protocol

**DOI:** 10.29337/ijsp.178

**Published:** 2022-08-01

**Authors:** Robertino B. Basso, Breyner Garcia Rodriguez, René M. Palacios Huatuco, Alejandro Coloccini, Mariano F. Ramírez, Horacio F. Mayer

**Affiliations:** 1 Plastic Surgery Department, Hospital Italiano de Buenos Aires, University of Buenos Aires School of Medicine, Hospital Italiano de Buenos Aires University Institute, Buenos Aires, Argentina

**Keywords:** Female, Mastectomy, Surgical flaps, Necrosis, Breast reconstruction

## Abstract

In recent decades, the mastectomy technique has undergone progressive adaptations in order to preserve the integrity of the breast structure and the nipple-areola complex (NAC), allowing reconstruction to be performed immediately after ablation. However, mastectomy flap necrosis or NAC has become a common complication, with an incidence between 2% and 22.3%. Blood glucose measurement to monitor microsurgical flaps has been reported as a simple method for the early detection of venous compromise. In this scenario, we propose the need to use an alternative, cost-effective method to assess the vitality of NAC in conservative oncological mastectomies. This protocol describes a prospective cohort study and was approved by the Research Protocols Ethics Committee of our institution. Patients will be included after signing informed consent. The anonymity and confidentiality of the information collected will be respected according to the Declaration of Helsinki and according to local and national guidelines.

**Highlights:**

- The rate of flap necrosis or nipple-areola complex (NAC) after mastectomy with reconstruction ranges from 2% to 22.3%.
- Glucose measurement has been reported in microsurgical flaps.
- This protocol seeks to determine the use of glucose as an early predictor of NAC necrosis.
- Participants will be recruited from a high-volume breast pathology hospital.

## Introduction

For a long time, conventional mastectomy without reconstruction was the surgery indicated for the oncological resection of breast cancer, neglecting cultural and psychological aspects of the patients and leading to delayed reconstruction, with aesthetic results that are more difficult to achieve. The rise of plastic surgery forced the modification of reconstructive planning after mastectomy and, with it, surgical techniques, facilitating immediate reconstruction [1]. Skin-sparing (SSM) and nipple-sparing (NSM) mastectomies are relatively new conservative surgical approaches to breast cancer. In SSM, most of the breast skin is conserved to create a pocket that facilitates immediate breast reconstruction with implant or autologous graft to achieve a quality cosmetic outcome. NSM is closely similar, except that the nipple-areola complex (NAC) is also conserved [2]. These advances made it possible to improve the aesthetic, psychological, and social aspects and the degrees of satisfaction of patients with lower hospital costs [3].

With these new skin preservation techniques, it became a challenge to perform oncological resection by dissecting between subcutaneous cellular tissue and mammary glandular tissue to obtain an adequate thickness of the flap and preserve its vitality. Wound complications, including flap necrosis, are the most common complications and can have a significant impact on both cosmetic results and costs. In a systematic review, 12358 NSMs were analyzed to assess complications and oncological safety. The overall rate of complication was 22.3% and the rate of necrosis of NAC was 5.9%. Several studies have reported data on NAC necrosis, with incidence rates ranging from 3.5% for total nipple necrosis to 12.1% for partial nipple necrosis [4]. The relationship between the increased rate of flap necrosis and other risk factors inherent to the patient has been reported, such as smoking, diabetes mellitus, high body mass index (BMI), previous radiotherapy, location of the incision, weight of the resected piece, among others.

Traditionally, clinical parameters such as skin color, capillary refill time, and dermal bleeding from the edges of the wound are used [5]. Currently, there are different methods used to assess the viability of the flap or the NAC [6]. A Stanford study used intraoperative skin perfusion assessment by laser-assisted indocyanine green angiography (SPY Elite) prior to mastectomy to identify perfusion patterns associated with ischemic complications of the NAC [7]. However, in Argentina, access to angiographic equipment represents a limitation, either due to its availability or costs.

The detection of poorly perfused tissue after mastectomy preserving NAC is determined according to the level of vascular compromise, from superficial epidermolysis that does not require intervention to full-thickness necrosis that is treated by debridement in the operating room [8]. Treatment of this complication can vary from local cures to debridement and the need to remove the prosthetic material. Although in most cases, the treatment is surgical and has certain repercussions with respect to reconstruction, aesthetic results, patient quality of life, and delays in adjuvant oncological treatments. Other complications that may occur due to poor flap perfusion can be infections, hematoma, and wound dehiscence.

Blood glucose measurement for monitoring microsurgical flaps has been reported as a simple method that can be widely used to decrease the rate of flap loss after tissue transplantation [9]. However, it is not a common practice in our environment.

Early detection of NAC necrosis would allow us to evaluate an intraoperative approach that involves its resection and avoid subsequent complications. In this scenario, we propose the need for the use of an alternative cost-effective method to assess the vitality of the NAC in conservative oncological mastectomies.

## Hypothesis

The decreased glucose ratio in the blood and the nipple-areola complex predicts NAC necrosis.

## Study Objectives

### Primary

Determine the usefulness of glucose monitoring in the early diagnosis of necrosis of the nipple-areola complex.

### Secondary

Determine the glucose cut-off point for the diagnosis of partial or total NAC necrosis.

Determine the diagnostic precision in early detection with respect to the clinic.

Determine whether it is a cost-effective tool for predicting complications.

## Materials and Methods

### Study Design

This protocol describes a single-center prospective cohort study. This research was approved by the Ethics Committee of Research Protocols of our institution, with No. 4240.

### Study population

All patients undergoing an oncological mastectomy and immediate breast reconstruction with tissue expander or definitive implant with preservation of NAC, from May 2022 to May 2025, operated by the Department of Plastic Surgery of the Hospital Italiano de Buenos Aires.

All patients will be informed through a personal explanation and brochures on the research project during their visit to our institution.

### Inclusion criteria

All women who are 18 years of age or older will be included in the study. Patients who undergo oncological mastectomy and immediate reconstruction with NAC conservation and those who agree and sign the informed consent document.

### Exclusion criteria

Patients under 18 years of age and men will be excluded. Patients without NAC conservation. Diabetic or insulin-resistant patients and those who cannot understand written informed consent.

## Sample size calculation

In total, our goal is to include 92 participants. The sample size was calculated in relation to the number of patients that make up the study population with a confidence interval of 95% and a margin of error of 5%.

## Studied parameters

Information about sex, age, BMI, comorbidities (obesity, dyslipidemia, high blood pressure, and tobacco use), ASA score, previous radiation or chemotherapy, tumor characteristics (size, location and histopathological type), degree of mammary ptosis according to Regnault’s classification [10], NAC thickness (mm). Furthermore, the type of surgical incision (hidden, vertical or transverse) [11], the weight of the mammary gland (g), the level of experience of the oncoplastic surgeon (Fellowship or senior), the operative time (min), the volume of prosthetic material (ml), the location of the flap and its complications (type of necrosis, wound infection, wound dehiscence, hematoma and seroma) will be recorded.

## Intervention and Considerations

### Glucose measurement method

The glucometer and the reactive strips “Accu-Check” will be used. Glucose measurements will be taken in the NAC and in the systemic blood. They will be performed on 3 occasions: during admission to the operating room (anesthetized patient without administration of hypo/hyperglycemic drugs), immediately after mastectomy and once breast reconstruction is complete (before skin closure). Measurements will be made in the area of the areola close to the incision or distal to its pedicle or free edge and another measurement in systemic blood. Additionally, a glucose measurement will be performed 7 days after surgery in the event of some degree of necrosis of the NAC.

### Follow-up

Controls to corroborate the vitality of the NAC will be performed with photos and clinical control (color, dermal bleeding, and capillary refill) at 24 hours, 7 and 14 days after surgery. Photographs will be taken from the front at a distance of 2 meters, and the patient will have both arms behind the back and hands together at the height of the buttocks, visualizing the patient from the shoulders to the navel.

## Statistic analysis

For the descriptive analysis, categorical variables were expressed as frequency (percentage) and continuous variables as mean ± standard deviation. All estimates will include confidence intervals. The Mann-Whitney test will be used for the comparison of continuous variables and the Chi-square test and Fisher’s exact test for the comparison of categorical variables. The ROC (Receiver Operating Characteristic) curves will be used as diagnostic tests to assess the diagnostic accuracy of the glucose ratio as a predictor of NAC necrosis, determining the sensitivity and specificity. The Youden index will be used to find the glucose cut-off point with the best diagnostic performance of NAC necrosis. Statistical analyzes will be performed with the Statistical Package for the Social Sciences (SPSS) version 26.0 (IBM Corp., Armonk, NY, USA) and a value of P < 0.05 will be considered significant.
